# A Focus on the Synergy of Radiomics and RNA Sequencing in Breast Cancer

**DOI:** 10.3390/ijms24087214

**Published:** 2023-04-13

**Authors:** Davide Bellini, Marika Milan, Antonella Bordin, Roberto Rizzi, Marco Rengo, Simone Vicini, Alessandro Onori, Iacopo Carbone, Elena De Falco

**Affiliations:** 1 Department of Radiological Sciences, Oncology and Pathology, I.C.O.T. Hospital, Sapienza University of Rome, Via Franco Faggiana 1668, 04100 Latina, Italy; 2 Department of Medical Surgical Sciences and Biotechnologies, Sapienza University of Rome, C.so della Repubblica 79, 04100 Latina, Italyelena.defalco@uniroma1.it (E.D.F.); 3 UOC Neurology, Fondazione Ca’Granda, Ospedale Maggiore Policlinico, Via F. Sforza, 28, 20122 Milan, Italy; 4 Mediterranea Cardiocentro, 80122 Napoli, Italy

**Keywords:** breast cancer, genomics, radiomics, radiogenomics

## Abstract

Radiological imaging is currently employed as the most effective technique for screening, diagnosis, and follow up of patients with breast cancer (BC), the most common type of tumor in women worldwide. However, the introduction of the omics sciences such as metabolomics, proteomics, and molecular genomics, have optimized the therapeutic path for patients and implementing novel information parallel to the mutational asset targetable by specific clinical treatments. Parallel to the “omics” clusters, radiological imaging has been gradually employed to generate a specific omics cluster termed “radiomics”. Radiomics is a novel advanced approach to imaging, extracting quantitative, and ideally, reproducible data from radiological images using sophisticated mathematical analysis, including disease-specific patterns, that could not be detected by the human eye. Along with radiomics, radiogenomics, defined as the integration of “radiology” and “genomics”, is an emerging field exploring the relationship between specific features extracted from radiological images and genetic or molecular traits of a particular disease to construct adequate predictive models. Accordingly, radiological characteristics of the tissue are supposed to mimic a defined genotype and phenotype and to better explore the heterogeneity and the dynamic evolution of the tumor over the time. Despite such improvements, we are still far from achieving approved and standardized protocols in clinical practice. Nevertheless, what can we learn by this emerging multidisciplinary clinical approach? This minireview provides a focused overview on the significance of radiomics integrated by RNA sequencing in BC. We will also discuss advances and future challenges of such radiomics-based approach.

## 1. Introduction

Breast cancer (BC) represents the most frequent neoplasia among women, with 2.1 million newly diagnosed every year. Thus, BC is the first common malignant cancer worldwide [1,2,3].

As strategies for cancer detection have largely improved [3] and, consequently, incidence rates have increased over the years, radiological approaches are still the first primary diagnostic work up in cancer with a key and essential role in diagnosis, staging, treatment planning, and response evaluation after therapy.

Technological advancement of radiological imaging has led to radiomics, which postulates that images obtained using magnetic resonance imaging (MRI), ultrasound (US), mammography, and digital breast tomosynthesis (DBT) contain more information that is not visible to the human eye and therefore underestimated. This information can be extracted using specific advanced software that can provide information regarding the texture of tissues of interest. 

Thus, the deep and simultaneous characterization of breast lesions throughout these techniques and advanced imaging analysis using artificial intelligence (AI) can be of utmost importance in the era of personalized medicine.

Separately, high-throughput molecular methods such as next-generation sequencing or RNA sequencing (RNAseq), are performed to deeply analyze the molecular features of BC tissue and to design the best suitable treatment for the patient. In particular, RNAseq has been useful to evaluate several aspects of BC, including biomarker selection, characterization of cancer heterogeneity, drug resistance, cancer immune microenvironment, and response to immunotherapy [3,4,5].

Nevertheless, great attention is currently being given to novel tools of prediction, where the abovementioned approaches (radiological imaging and molecular mutational analysis, metabolomics, or proteomics of the targeted tissue), often considered diametrically opposed, are combined [6].

This emerging strategy, defined as radiogenomics, assumes that extracted imaging data are the product of mechanisms occurring at a genetic and molecular level linked to the genotypic and phenotypic characteristics of the tissue. In other words, radiogenomics is based on the idea that genetic changes in tumor biology control radiologic phenotypes [7,8].

Despite this, the development of predictive models of radiogenomics to be fully employed at the clinical level, is still a challenge. These drawbacks will be cited later in this review.

Among several molecular-based approaches to defining tumors and to be integrated into radiogenomics, the RNA-seq-based strategy appears as the most versatile and useful to describe the phenotype of highly heterogenous types of cancer such as BC. Thus, the transcriptomic profile is considered a key feature enabling the characterization of the whole tumor’s ecosystem including specific cell subpopulations, microenvironment, and T cell profile [9] and with added clinical utility [10,11].

In this minireview, the most relevant biomarkers obtained from the prevalent RNAseq technologies and advanced imaging analyses are summarized, and their applications in BC and future challenges and opportunities are discussed.

## 2. Artificial Intelligence and Radiomics to Improve Detection of Breast Lesions and Response to Treatment

Large-scale screening programs represent the best strategy to diagnose BC in the early phase, leading to more therapeutical opportunities and favorable prognostic implications. Currently, more than 42 million mammographic examinations are performed in the USA and UK for this purpose.

The increasing number of clinical exams performed every year has led to developing several strategies to improve a faster and more effective approach to interpreting radiological images. In this regard, a first attempt emerged in the 1990s, when the first computer-aided-diagnosis (CAD) systems were developed [12,13,14], setting the basis for the application of AI software into clinical practice.

Since then, continuous efforts in the AI field have succeeded in developing cutting-edge AI systems as an interesting opportunity to screen patients more efficiently, improving both the sensitivity and specificity of the exam, therefore, potentially increasing the rate of early detection and reducing recalls and unnecessary second-level examinations. 

Several authors have already demonstrated the potential role of AI to foster the early detection of BC. Rodriguez-Ruiz [12] and coworkers compared BC detection performance of radiologists reading mammographic examinations compared to those supported by AI systems, showing that diagnostic performance increased with AI-based technology, with higher rates of sensitivity and specificity, and increased area under the curve (AUC) values. This result was corroborated by a second study of the same group, where the AI system was compared to 101 breast radiologists to evaluate mammograms for BC identification: the AI system achieved an AUC of 0.84, not inferior to the average of radiologists [12].

Moreover, McKinney and colleagues presented an AI deep learning model for identifying breast cancer in screening mammograms using two large datasets from the UK and the US. The model outperformed radiologists in terms of assessment of individual lesions and individual breasts, obtaining an AUC of 0.79 compared to that of 0.74 reached by 104 radiologists [15].

Similar studies have also strengthened the role of AI in the analysis of DBT to provide a three-dimensional (3D) model reconstruction system for breast tumors starting from a small number of mammograms. 

AI algorithms have also been employed to improve the potential of second-level imaging techniques, such as MRI. Even though the latter has the highest sensitivity in BC detection, its role is limited by its availability, cost, and long acquisition time [16,17].

To overcome these drawbacks, Dalmiş and colleagues developed a new AI-CAD system for breast MRI using only the early phase of the acquisition with 3D sequences, able to detect 60% more lesions compared to standard protocols, paving the way to apply “fast MRI protocols” in BC screening [18].

The use of AI combined with radiomics may also be a further strategy to guide clinical decision-making during the setting of treatment choice. Thanks to its quantitative approach, radiomics enables the objective assessment of relevant intrinsic imaging features, such as those showing tumor heterogeneity. Therefore, radiomics may help to improve the objectivity and accuracy of treatment response, where the imaging features may be used together with other forms of data in predictive modeling [19,20,21].

For example, Tahmassebi et al. [22] used a machine learning (ML) algorithm based on multiparametric features obtained from an MRI of the breast to predict pathological complete response to neoadjuvant chemotherapy and survival outcomes in BC patients. The algorithm obtained allowed us to evaluate the histopathologic residual cancer burden (AUC: 0.86) and recurrence-free survival rate (AUC: 0.83).

Similarly, convolutional neural networks (CNNs) have been recently developed to predict response to treatment, such as the one studied by Ha et al. [23] in a retrospective study to evaluate if CNN applied to pre-treatment breast MRI images could be used to predict neoadjuvant chemotherapy response, achieving an overall accuracy of 88%. 

Moreover, D’Angelo et al. [24] demonstrated that an automated breast volume scanner may be a useful method to detect BC patients who are more prone to reach a complete pathological response during neoadjuvant therapy: in a cohort of 21 patients, the authors compared an automated breast volume scanner to manual hand-held US and contrast-enhanced MRI, with the former being more reliable than manual US, and comparable to MRI.

In clinical practice, many promising algorithms may not function as reported in the literature. However, these studies advocate the potential of AI-based informatics tools on treatment outcomes predictions, encouraging researchers to test and implement new and existing algorithms in practice, with the aim of improving BC clinical management.

Traditionally, radiomics features provide information regarding grey-scale patterns, inter-pixel relationships, shape, and spectral properties within regions of interest (ROIs) on radiological images.

Radiomics analysis is an analytical framework applicable to various target sites, mainly tumors, and imaging modalities, including US, computed tomography (CT), MRI, and positron emission tomography (PET). In particular, radiomics has become a potential method for the non-invasive evaluation of the patient’s disease, enabling the tumor’s spatial heterogeneity and its temporal alterations through investigation of the tumoral microenvironment.

The first step is the acquisition of suitable images. In BC, MRI, US, mammography, and DBT represent the main radiological tests. Due to the variability of different instrumentations, acquisition parameters, and operators across radiological laboratories, standardized imaging protocols are necessary and recommended to perform an accurate quantification of imaging, in order to improve reproducibility and applicability of radiomics features [25].

**Figure 1:**
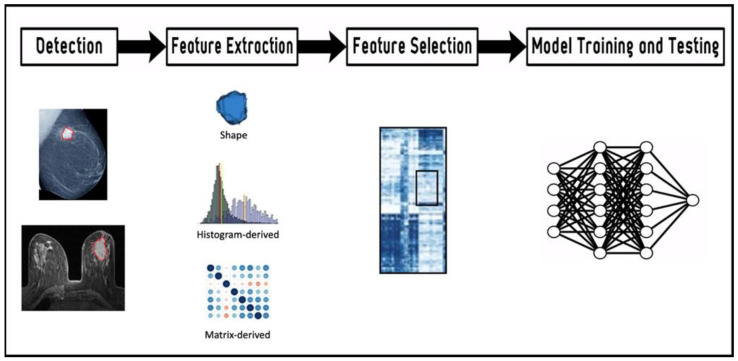
Workflow of the radiomics features extraction and analysis process. Lesions detected by imaging (mammography and magnetic resonance imaging) are contoured in red.

The second step is the definition of an ROI within the lesion from which radiomics characteristics are computed. The third step is featuring extraction using mathematical formulas and AI algorithms, broadly classified in four categories: morphological, histogram-based, textural, and transform-based features. Hundreds of thousands of features are computed, exhibiting varying degrees of complexity and expressing properties of the lesion morphology and the voxel intensity histogram, as well as the spatial organization of the intensity values at the voxel level (texture). During the selection process, only the features related to specific clinical questions are considered. The concept underlying the process is that radiological images contain qualitative and quantitative information, which may reflect the underlying pathophysiology of a tissue. Artificial intelligence algorithms play a role in this process, selecting the relevant features. Indeed, feature selection seeks to identify which of these features are stable, non-redundant, and/or robust to any intrinsic dependencies (e.g., shape, volume) [26]. Next, models are built employing the selected features according to the clinical queries. Throughout this workflow (Figure 1), imaging biomarkers can be extracted and used for diagnosis but also for predicting treatment response and risk of recurrence.

## 3. RNA Sequencing in Breast Cancer

The RNAseq technologies have a crucial application in oncology for the discovery of key genes and signaling pathways, which can be employed as biomarkers signatures for cancer diagnosis, prognosis, prediction, and as therapeutic targets [27]. The prevalent RNAseq technologies suitable in breast oncology are bulk RNAseq, laser capture micro-dissected RNAseq (LCM), single cell RNAseq (scRNAseq), GeoMX digital spatial profiling (DSP), and spatial transcriptomic (ST).

Among all BC, the triple negative breast cancer (TNBC), the most aggressive and malignant tumor due to its high heterogeneity in distinct molecular subtypes [3,28,29], offers the best example where the majority of studies regarding the integration of specific transcriptomic and radiomics is directing compared to other BC types. The TNBC is characterized by a poorer prognosis, high rate of relapse, and metastasis leading to tumor recurrence [3,30]. RNAseq-based technology has been developed to provide an average of aberrantly expressed mRNAs in samples. This approach has made possible the classification of TNBC into four different subtypes: basal-like immune-activated (BLIA), basal-like immune-suppressed (BLIS), mesenchymal (MES), and luminal androgen receptor (LAR) [31]. The discovery and implementation of new drugs targeting biomarkers is essential to highlight the mechanisms of resistance that result in poorer prognosis for TNBC patients. The TNBC stratification allows us to identify subtype-specific prognosis signatures, improving the development of personalized therapy. The infiltration of immune cells into tumors varies in TNBC subtypes, leading to different responses to chemotherapy [32]. For example, Filho et al. discovered that the addition of carboplatin to standard neoadjuvant chemotherapy has beneficial effects against tumors with higher inferred CD8+ T-cell infiltration [33].

When the RNAseq analysis of TNBC is compared to that exhibited by normal tissues, nine steroid hormone-related genes (*FSIP1, ADCY5, FSD1, HMSD, CMTM5, AFF3, CYP2A7, ATP1A2*, and *C11orf86*) can be identified and significantly associated with the prognosis. The abovementioned markers can be regarded as putative prognostic markers, confirming the role of hormones in TNBC tumorigenesis [34]. In 2019, Jiang and colleagues analyzed eight clinical specimens of BC and paracancerous breast tissues through transcriptomic sequencing and found that six differential expressed genes are upregulated (*CST2, DRP2, CLEC5A, SCD, KIAA1211, DTL*), whereas a different set of six genes were found downregulated (*STAC2, BTNL9, CA4, CD300LG, GPIHBP1*, and *PIGR*) although differences for RFS and OS were not highlighted. Data obtained from RNA-seq underline the difference between TNBC tissues with or without distant metastasis. Khaled et al. founded a group of differentially expressed genes involved in the regulation of cell–cell adhesion, immune-modulation, and Wnt/β-catenin pathways [35]. Specifically, the researchers discovered that overexpression of *SHISA3* controls the epithelial-mesenchymal transition in TNBC, inhibiting cell proliferation and invasion. The results suggest that *SHISA3* could represent a novel tumor suppressor gene and a possible therapeutic target for TNBC treatment [35].

When the computational analysis of the RNA transcripts in BC is performed by employing databases such as the KEGG database, (which highlights specific signaling pathways and molecular effects on targeted proteins), an enrichment of pathways related to the extracellular matrix-receptor (ECM-receptor) interaction, including genes of the tetraspanin family, collagen, and fibronectin can be found [36]. Tetraspanins are transmembrane transport proteins except for TSPAN15, which is connected to the NOTCH signaling system [37,38]. Specifically, the expression of TSPAN1 has been found to be higher in BC tissue and associated to positivity to the estrogen receptor (ER) and the human epidermal growth factor receptor 2 (HER2) [39].

Additional differential expressed genes in TNBC have been identified by Zhang and colleagues through the integration of several bioinformatics research methods [40]. In this study, out of 1060 genes screened in both breast and healthy samples, 544 genes were up-regulated and 516 were down-regulated in the cancer tissue. In particular, the authors have narrowed the field, focusing on 23 genes as potential diagnostic markers. These genes are involved in many cancer-related biological processes and pathways, such as chemical carcinogenesis, drug metabolism, xenobiotic metabolic process, metabolic pathways, and oxygen binding. Some of these 23 genes have been confirmed to be related to BC in literature, including *ADH1A, ADH1C, AKR1C4, ALDH3A1, CYP1A2, CYP2B6, CYP2C18, CYP2C19, CYP3A4, CYP3A7, GSTA1*, and the *RXRG* gene [40,41].

The analysis of the transcriptomic profile cannot be limited to the sole cancer tissue. The TNBC is acknowledged as one of the most heterogeneous types of tumor with markedly biological and different microenvironments generated within the tissue [3,42]. This heterogeneous biological “cancer ecosystem” is mainly characterized by a plethora of cell components such as epithelial cells, invasive, or in situ tumor cells, surrounding stroma, infiltrating immune cells, blood vessels, and capillaries, which constitute the tumor microenvironment (TME) [3,43]. Therefore, the analysis of the RNAseq, restricted to a single cell population rather than the whole TME, could misrepresent the driver molecular target/mutation and be responsible for drug resistance, if not properly identified. Indeed, stromal tumor-infiltrating lymphocytes (TIL) are associated with good prognosis in patients with TNBC, being able to predict chemotherapy response [44].

One of the technical approaches that may overcome the limitation of bulk RNAseq, is the laser capture microdissection (LCM), by which each cell’s population, in formalin-fixed paraffin-embedded breast cancer samples, are collected separately based on their different morphology. The integration of spatial distribution of immune cells with the laser capture microdissection and the gene expression profiles of tumor stroma and epithelium compartments, has resulted in an improved stratification of tumor immune microenvironment (TIME) [45], providing tools for the optimization of immunotherapy and identification of new biomarkers. TIME subtypes have distinct immune landscapes and potential escape strategies that involve different patterns of immune checkpoint proteins (PDL-1 and B7-H4), immune modulators (IDO1), immunomodulatory cell type infiltration (macrophages, Tregs, neutrophils, and IL-17-producing cells), and HLA-I loss [46].

The next generation of scRNA-seq technology has made it possible to improve the understanding of the molecular processes that underlie the biological behavior of BC. The heterogeneity, dynamic growth, and differentiation process of single cells are shown by scRNA-seq analysis of cell phenotypes and transcriptome variations. This approach has led to the identification of three different epithelial cell populations: basal (KRT14^+^), secretory luminal1 (KRT18^+^/SLPI^+^), and hormone-reactive luminal2 (KRT18+/ANKRD30A^+^) cell types, which can be linked to several breast tumor subtypes [47]. Moreover, high expression levels of genes related to cancer stem cells (CSC) are found in patients with high risk of recurrence, suggesting that they can be used as putative BC biomarkers [48]. Different cell subpopulations of cancer-associated fibroblasts (CAFs) located in the microenvironment have been identified through the scRNA-seq in a mouse model of BC (MMTV-PyMT). The authors divided CAFs according to the marker genes of their histological localization: vascular CAFs (vCAFs), matrix CAFs (mCAFs), cycling CAFs (cCAFs), and developmental CAFs (dCAFs). Each subtype has a specific function; for instance, vCAFs or mCAFs are indicators of a tumor’s propensity to metastasize [49]. The development of scRNA-seq technology offers greater opportunities for a deep investigation of drug resistance mechanisms and a more precise interpretation of transcriptome data. A comprehensive transcriptional atlas of the cellular architecture of BC has been published in 2021 [50]. In this work, the authors identified recurrent neoplastic cell heterogeneity of BC, developing a single cell method of intrinsic subtype classification (scSubtype). The Cellular Indexing of Transcriptomes and Epitopes by Sequencing (CITE-Seq) enabled the identification of a new PD-L1/PD-L2+ macrophage groups linked to clinical outcome. CITE-Seq is a sequencing-based method that simultaneously quantifies cell surface protein and transcriptomic data within a single cell readout. Among thousands of primary BC cases, nine ecotypes were identified. Interestingly, nine-ecotype clustering is driven by cells from all three major lineages (epithelial, immune, and stromal).

Furthermore, single-cell transcriptional analysis of 32 breast cell lines demonstrated that this approach is helpful to discover clinically important clinical markers. HER2 expression in the MDA-MB-361 cell line showed a dynamic plasticity that could affect drug response. This phenomenon was also observed in circulating tumor cells of BC [51].

The GeoMx Digital Spatial Profiler (DSP) enable researchers and doctors to the discovery of spatial molecular signatures through the identification of proteins and RNA transcripts in the distinct regions of the tumor and surrounding tissues that are associated with disease progression, immune evasion, metastasis, and drug resistance [52]. Carter and colleagues, using GeoMx, evaluated the immune response to BC and find potential treatment targets in a cohort of untreated PDL-1^+^ and PD-L1 TNBC [53]. The spatial transcriptomics (ST) of eight HER2-positive BC [54], revealed the presence of tertiary lymphoid-like structures and a Type I interferon response that overlapped with areas of colocalization of macrophage and T-cell subsets [55]. The ST technology has a better throughput than digital spatial profiling techniques and does not require specialist equipment or prior knowledge of gene sequences. These latest technologies will certainly improve the precision oncology. A summary of the strengths and limitations of the abovementioned RNAseq-based approaches is listed in Table 1.

## 4. Matching Molecular and Radiological Features to Enhance Characterization of Breast Lesions

Breast lesions can be characterized by imaging techniques according to their typical radiological features. The application of radiogenomics in BC, revealing its potential as a promising tool to improve diagnosis and to develop new therapeutic strategies for BC treatment although it has to be validated in larger cohorts of patients for a full clinical application [55]. Patterns of breast calcifications visible on mammograms may be useful to differentiate benign and malignant lesions. In 2012 Yamamoto and colleagues were the first authors to identify 21 MRI features, correlated with 71% of gene expression profiles [57]. In a different study, the same researchers discovered that the increasing rim fraction score was related to the expression of long non-coding RNA linked to metastasis-free survival in 70 patients with BC [58]. Radiological features of the breasts have been already used by Cai et al. [59] to create a deep learning (DL)-based CNN capable of discriminating among benign and malignant microcalcifications.

Similarly, breast lesions feature visible on MRI such as shape, margins, calcification morphology, mammographic breast density, and enhancement patterns are considered in Descriptive Breast Imaging Reporting and Data System (BI-RADS) to characterize BC. These features have the potential to act as imaging biomarkers of BC recurrence risk and may provide guidance for specific treatment approaches [60]. Zhou et al. [61] developed a CNN model based on T1-weighted MRI images to generate a 3D mask of the breast area, achieving the highest sensitivity for BI-RADS 5 (92.5%), and a low value for BI-RADS 3 (33.3%), indicating that BI-RADS 3 represents an uncertain category not only for radiologists, but also for DL approaches. However, their results showed that the model could serve as an assisting tool in the report system to help raise specificity in cancer screening.

To date, the characterization of BC lesions is not limited to the radiological assessment, but imaging is more frequently associated with molecular investigations of the genomic profile of the patient. It is acknowledged that the tumor genomic profile plays a crucial role in the characterization of BC both for implications related to the somatic mutations of the lesion or to the expression of an unstable or pathogenic genotype with hereditable characteristics [3].

Matching imaging and molecular mutational analysis exhibited by the subject, defined as radiogenomics, is still a challenge. Nevertheless, several studies regarding the beneficial effects of such strategy, are emerging.

The growing field of radiogenomics, with its potential to interrogate data from imaging appears to be promising in outlining diagnostic information when accompanied to molecular features of BC. Several studies have been published on this topic. Novel computer extracted mammographic texture features (AVE, MinCDF, Energy, MaxF (COOC)) have been elaborated to distinguish BRCA1/2 mutation carriers from non-carriers. Specifically, the Energy and MaxF (COOC) characteristics indicate the spatial distribution pattern for tissue homogeneity, whereas AVE and MinCDF provide information about the tissue density. Patients with BRCA1/2 mutation are characterized by retro-areolar parenchymal patterns whit coarse texture [62]. The identification of BRCA gene mutations, predicts which women have a high risk in developing BC [63]. MRI-based parameters could also identify BC with over-expression of the human epidermal growth factor receptor 2 (HER-2). To date, its detection mainly depends on invasive tissue biopsy. Zhou et. al. [61] developed interesting radiomics models to predict HER-2 gene status (which relates to a more aggressive subtype of BC), therefore providing a novel tool to support clinical decision-making, and attempting to overcome some limitations of the fluorescence in situ hybridization and immunohistochemistry of HER-2, especially those regarding the representativeness of the bioptic sample when withdrawn by the patient.

Performance of these models was tested with attractive results: they obtained an AUC of 0.85 in the training set, and an AUC of 0.79 in the testing set, suggesting that radiomic features extracted from mammograms may be a non-invasive method for pre-operative evaluation of HER-2 status in BC.

Zhang et al. [64] demonstrated the potential role of a radiomic features derived from apparent diffusion coefficient (ADC) maps of breast MRI in patients with invasive ductal BC that may be used as a pre-operative predictor of Ki-67 index, since it resulted in the ability to differentiate the low and high Ki-67 index with high performance (AUC: 0.75). Li and colleagues elaborated a functional parametric map of DCE-MRI, to classify the HER2 and Ki67 expression in BC, through the characterization of both intratumoral and peritumoral regions [65]. 

Radiogenomics also allows the classification of BC molecular subtypes namely Luminal A, luminal B, HER-2 enriched, and basal-like, through the combination of specific miRNAs with imaging features of each BC subtype [66], generating specific radiomiRNomic maps. The tumor progression and the heterogeneity of the blood vessel system are regulated by miRNAs [67]. An encouraging correlation has emerged between BC with spherical and less irregular characteristics, obtained from Dynamic Contrast Enhanced Magnetic Resonance (DCE-MRI), and an increase of the apoptosis genes expression [68]. The size and morphologic radiomics features such as texture, diameter, and perimeter, derived from DCE-MRI have been demonstrated to provide information about the metastatic capacity of BC and the composition of the microenvironment, in particular neutrophils, fibroblasts, and endothelial cells in a small cohort of patients [69]. The DCE-MRI is a high-performance modality to individuate genomic biomarkers, including cell cycle check points, genes such as *Myc, PI3K, RTK/RAS, P53, and ER + /ER−, PR + /PR−, HER2 + /HER2,* and triple-negative indicators [70]. Bismeijer and colleagues established that the tumor size changes in concomitant with the proliferative rate of the mass [71]. The authors were able to recognize seven MRI factors: tumor size, shape, initial enhancement, late enhancement, smoothness of enhancement, sharpness, and sharpness variation. They discovered that the expression of ribosomal proteins required for ribogenesis and regulated by the mammalian target of rapamycin or mTOR pathway, is linked to low initial enhancement, increased smoothness, and low sharpness. In the field of medical oncology, anticancer drugs which target the mTOR pathway are currently being developed. Given these findings, it is possible that in the future, patient candidates for therapy targeting mTOR will be identified based on MRI features of BC, enhancing kinetics and tumor boundaries. Moreover, the expression of genes related to extracellular matrix and collagen synthesis are associated with an irregular shape of the tumor, highlighting the central role of fibroblasts in BC development [71]. 

The combination of OncotypeDX and PAM50 gene panels (which provide a prediction score of temporal recurrence in subjects with estrogen-receptor-positive, HER2^-^ BC) [72] and imaging phenotype trough radiomics has been demonstrated to strengthen the prediction significance of the above-mentioned molecular profiles [73,74]. 

Radiomics is a non-invasive approach which can also identify and characterize the different TNBC molecular subtypes, analyzing the spatial domain features and sequential feature obtained by Contrast-Enhanced Magnetic Resonance Imaging (CE-MRI) [56]. Therefore, radiogenomics, in predicting gene mutations, plays a crucial role in the early detection of various BC subtypes, preventing patients from undergoing invasive procedures.

Moreover, radiogenomics, in the radiotherapy field, aims to find indicators which can predict the sensitivity of tumor and healthy tissues to radiation in order to develop a more personalized therapy [70]. PET imaging of 18F-fluorodeoxygucose (FDG) is frequently used to mark tumors in order to determine its diffusion in the body and assess the response to a treatment, although the FDG uptake is still not fully understood [75]. Recently, Ralli and colleagues have identified enriched pathways significantly associated with the uptake of FDG in BC, such us glycolysis/gluconeogenesis (GLYC-GLUC) and many immune-related pathways [76].

To date, there are different hardware or scanning techniques and the standardization of images and normalization is crucial for the radiogenomics of the future [8]. 

Recently, cancer research has focused on investigating the peritumoral area surrounding the cancer mass. The peritumoral region gives biological key information about angiogenesis, lymphangiogenic activity, metastatic invasion of lymphatics and blood vessels, as well as immune response within peripheral BC, such as stromal response and lymphocytic infiltration. All these features are putative biomarkers [65]. Liu et al. [61] validated an AI model based on radiomic features extracted from BC on contrast-enhanced MRI images to predict sentinel lymph node metastasis, with a sensitivity in detecting positive sentinel lymph node of 71%, and an AUC of 0.83.

In conclusion, BC parameters obtained from MRI, can predict the underlying genes expression emerged by the RNAseq. Systemic reviews and multiple studies on small cohort of patients are suggesting that the integration of molecular, anatomical, and functional approaches in radiogenomics, might allow us to classify significant features of tumor tissue, that could become potential biomarkers for the development of personalized medicine. Specifically, radiogenomics and/or radiomics could assist in the early detection and classification of cancers, differential diagnosis, mapping BC before surgery, correlation between imaging features and tumor molecular biomarkers, prediction of metastasis, and chemotherapy benefit prediction [33,70,77,78,79,80].

Mentioned biomarkers and their functions are summarized in Table 2.

## 5. Conclusions

Radiogenomics in BC has the potential to integrate the clinicopathological data trough imaging biomarkers and prognostic gene panels. This strategy will help to improve the understanding of the tumor heterogeneity, to predict the potential evolution of the tissue and to enhance the stratification of patients, helping to design tailored clinical treatment in the era of personalized medicine.

So far, the application of the proposed radiogenomics approaches in real clinical practice is still hampered by the several concerns, therefore limiting the role of the radiogenomics to research purposes by mostly reconstructing predictive models with a potential use to develop individualized treatments to ultimately affect patients’ prognosis [81,82]. In BC, the radiological imaging and the plethora of “omics” science would represent a significant improvement for patients if combined with genomic data, which have been already strictly correlated with the prognosis [83]. To date, a mix of semiautomatic and fully automatic software to quantify imaging coexists [84,85], providing potential different interpretation among laboratories and a range of non-standardized and unvalidated protocols. Specific criteria of acquisition have not been homogenously identified and acknowledged as well as guidelines which may help to harmonize and convert algorithms and omics in precise clinical scores.

Notably, all omics-based data represent a precise photograph of a defined phase of the cancer disease. Thus, the development of standardized protocols should consider that the match with radiological imaging should be performed in parallel or consecutively in a time window. In the light of this, the huge amount of clinical imaging data that can be derived from the single patient in a fixed interval of time (normally ranging from diagnosis to follow ups), will necessarily require future automated programs which may even reconstruct the whole radiological scenario, highlighting significant changes of the tissue over the time. Accordingly, among all tissue characteristics highlighted by radiomics, the dynamic features (from tissue texture to uptake of tumors) are likely to be the most promising criteria, as they can be better associated to molecular data and to those obtained from specific regions of interest such as lymph nodes or area of metastasis, where different genomic alterations frequently occur.

In addition, there could be some potential for AI-supported clinical management. However, the algorithm developing process is still in progress, and there is a need to connect multi-center projects. Indeed, radiogenomics is still the main research domain, with thousands of mostly retrospective single-center studies. Both long term and large scale clinical radiogenomics data are missing, and the results obtained from the omics profiles have not been yet associated with radiomics. Accordingly, a larger amount of data and prospective clinical trials that validate radiogenomics signatures on external datasets, are needed to improve the clinical workflow and demonstrate a real benefit for patients.

Moreover, an interdisciplinary-based approach, which could combine the knowledge and insight of radiologists, pathologists, oncologists, data scientists, and patients themselves is urgently required, to accelerate radiogenomics as a clinical diagnostic and predictive tool.

Cost-benefit ratio is still a real concern and further research is still needed to make this new approach feasible [86]. Notably, additional economic drawbacks derive from a high level and specific training for the personnel and high-throughput technologies imputed into all hospitals. Accordingly, to assess AI’s genuine value and capability to produce desired results, healthcare facilities must be meticulous in their cost-benefit analyses, as with any emerging technology. Although AI might offer advantages in improving workflow efficiency and clinical results in the future, its employment should be constantly tested against the current standard of care to ensure an additional advantage in terms of savings costs.

To date, improvements of a radiogenomics-based approach are exploiting alternative basic biological hallmarks of tumors, such as angiogenesis. Intriguingly, RNA-sequencing of a small cohort of BC has been associated to morphological and vascular imaging, revealing that a group of differentially expressed genes relating to dysfunctional alterations of the microvasculature, are also linked to poor histological grade and invasiveness [11], suggesting novel therapeutic molecular targets.

Additional data derived from radiogenomics, are being employed to evaluate the predictive efficacy of the neoadjuvant chemo or radiotherapy, which cannot be assumed as a homogenous treatment for all patients with BC, as it is well acknowledged that women can respond differently, even with the same histological grade and molecular profile.

Some protocols related to radiogenomics such as GEENEPI (genetic pathways for the European Society of Radiotherapy and Oncology) [87] represent a significant example to discriminate the effect of radiotherapy among cancer and peritumoral tissue. However, we are still far from quantifying a clinical index of response to therapy based on radiogenomics.

Other important questions remain open and still unexplored: is it feasible to apply radiogenomics to liquid biopsy as both are considered less invasive techniques? Besides, it would be also useful to match molecular techniques to radiomics according to the timing of the analysis: for instance, at diagnosis radiomics might be matched to the genomic profile of the primary BC, whereas metastatic areas should be separately analyzed and quantified acquiring new radiological imaging parallel to a further molecular profile of the metastatic tissue involved.

Finally, what else can radiogenomics predict? Other radiological criteria and models are not necessarily linked to mechanic characteristics or stiffness of the BC tissue and could add novel information at diagnosis or even during the screening, leading clinicians towards a better personalized medicine by, for instance, developing novel predictive algorithms and mainly by improving the very low number of experimental clinical trials. Accordingly, only two clinical trials have been specifically designed in BC and currently recruiting to evaluate DNA polymorphisms for Predicting the Effects of Radiotherapy (RAPPER) and a second study which investigates how radiogenomics can predict the pathologic response of pre- and post-menopausal women with BC in presence of genetic alterations (www.clinicaltrials.gov, accessed on 5 April 2023). A further improvement could be represented by the stratification of patients which can be potentially better achieved by the integration of radiogenomics with the current clinical practice. In fact, key biological differences among subjects, including resistance to therapy, could be better identified and discriminated among patients who can really benefit in the presence of equal treatments. According to this premis, it could be possible to construct high-fidelity models over the time that are likely to improve the mimicking of cancer biology rather than a model based only on genomic biomarkers or single radiological imaging.

## Figures and Tables

**Table 1:** RNAseq-based approaches: strengths and weaknesses.

Technique	Description	Strenghts	Weaknesses	References
Bulk RNAseq	Global gene expression profile of tumor sample	Identification of putative prognostic markers	Less sensitivity, loss of tumor heterogeneity evaluation	[34,36,37,40,41,56]
Laser Capture Micro-Dissected RNAseq (LCM)	Transcriptomic profiling of single tumor cell populations	Focus on the cellular heterogeneity of the tumor	Limited quality/quantity of RNA	[28,29]
Single Cell RNAseq (scRNAseq)	Investigation of RNA transcripts within individual cells	Highly sensitive	High cost, absence of analysis of the spatial tumor complexity	[47,48,49,50,51]
GeoMX Digital Spatial Profiling (DSP)	Spatial gene expression profile of formalin-fixed paraffin-embedded tissues	Characterization of tumor microenvironment	Limited to small number of genes	[52,53]
Spatial Transcriptomic (ST)	Spatial sequencing analysis	Characterization tumor microenvironment, supported by sequencing data	Limitations in microarray spot size and spacing; lack of single cell resolution	[54,55]

**Table 2:** Putative biomarkers and relative functions.

Biomarkers	Method	Functions	References
Texture features: tissue density and homogeneity.	DCE-MRI	BRAC1/2 patients’ classification; Early detection of BC subtypes: Luminal A, luminal B, HER-2 enriched and basal-like); Correlation with aberrantly expressed miRNA to generate radiomiRNomic maps; Personalized treatment planning.	[63,65,67,73,74]
Calcification morphology.	DCE-MRI	Discrimination between benign and malignant lesions.	[59]
Tumor size, shape, smoothness, sharpness variation; late enhancement.	DCE-MRI	Identification of specific altered pathways (e.g., mTOR; EMT, collagen synthesis).	[70,71]
Peritumoral area characterization.	CE-MRI	Assessment of metastatic invasion; Lymphocytic infiltration size.	[65]
FDG uptake.	PET	Determination of tumor diffusion in the body; Evaluation of treatment response.	[75,76]

## Data Availability

Not applicable.
